# Progressing Towards Strengthened Vaccination Programmes: Investigating COVID-19 Vaccine Usage in Public and Private Sectors in KwaZulu-Natal, South Africa

**DOI:** 10.3390/vaccines13111143

**Published:** 2025-11-07

**Authors:** Viloshini Krishna Manickum, Lehlohonolo John Mathibe

**Affiliations:** Discipline of Pharmaceutical Sciences, School of Health Sciences, University of KwaZulu-Natal, Durban 4041, South Africa; mathibel@ukzn.ac.za

**Keywords:** COVID-19, vaccine usage rates, stock visibility systems, electronic vaccination data systems, health care workers’ perspectives, monitoring, evaluation

## Abstract

**Background/Objectives:** Vaccine usage rates (VURs) for COVID-19 vaccines (C19V) in KwaZulu-Natal (KZN), South Africa, remain insufficiently documented. This study assessed VUR for Pfizer–BioNTech (Pfizer) and Janssen Biotech Inc. (J&J) and reviewed monitoring systems in public (PUBS) and private (PRIVS) sectors. **Methods:** A dual-phase, multicentre study was conducted in PUBS and PRIVS facilities. Phase 1 comprised a retrospective, cross-sectional analysis of VUR for Pfizer and J&J (May 2021–July 2022). Phase 2 involved qualitative interviews with public (PUBSR) and private (PRIVSR) sector respondents (January–March 2024). **Results:** Pfizer VURs were 78.7% (PUBS) and 104.4% (PRIVS), while J&J recorded 64.7% (PUBS) and 40.2% (PRIVS). Stock reconciliations were complete across PUBSR and PRIVSR, but challenges persisted in stock on hand, reporting systems, and operational indicators. **Conclusions:** Pfizer achieved higher VURs than J&J, with PRIVS exceeding 100% due to under-reporting of issued doses. Integrated, real-time monitoring of VURs is urgently required to strengthen evidence-based policymaking, optimise supply chain management, reduce wastage, and improve vaccine uptake. Standardised monitoring frameworks across PUBS and PRIVS are essential to align with national objectives.

## 1. Definitions

Vaccine usage rate refers to the number of doses administered divided by the number of doses issued [1].

Vaccine uptake rate refers to the number of people who receive a specific dose of the vaccine within a certain time period, which can be expressed either as an absolute number or as a proportion of the target population [2].

## 2. Introduction

The COVID-19 (C19) outbreak, first reported in December 2019 in Wuhan, China, was declared a global pandemic by March 2020 [3]. By December 2020, KwaZulu-Natal (KZN), the second-most populous province in South Africa (SA), had reported 194,629 confirmed cases and 4278 deaths [4]. In early 2021, several countries, including SA, procured COVID-19 vaccines (C19V) [5]. The National Department of Health (NDOH) aimed to vaccinate 67% of the population [6], expanding access through both public (PUBS) and private (PRIVS) facilities. The phased C19V programme began in February 2021 with the Sisonke study targeting healthcare workers, followed by general population rollout later that year [7].

By December 2023, 67% of the global population (≈5.4 billion people; 13.6 billion doses administered) had completed a primary vaccination series, compared with only 33% in Africa (≈646.5 million) and 35% in South Africa (≈41.8 million) [8]. In KZN, 4.93 million doses were administered by March 2022-, although provincial vaccination rates remain inaccessible [9]. These figures highlight global inequities, with Africa and SA lagging in coverage [10].

Monitoring vaccine usage rate (VUR)—the proportion of doses administered relative to those issued—is critical for identifying inefficiencies and reducing wastage [1]. While other countries have demonstrated improvements in coverage through supply chain redesign [11,12,13], no published studies have reported COVID-19 VURs in South Africa.

This study, therefore, investigated VUR for Pfizer–BioNTech (Pfizer) and Janssen Biotech Inc. (J&J) and reviewed monitoring systems in PUBS and PRIVS facilities in KZN, South Africa.

## 3. Methods

### 3.1. Study Design

This mixed-methods, dual-phase, multicentre study was conducted at public (PUBS) and private (PRIVS) healthcare facilities in KZN, SA. Phase 1 constituted a quantitative, retrospective, cross-sectional, observational study. Phase 2 was a qualitative, interview-based study in which public sector (PUBSR) and private sector (PRIVSR) vaccination managers/respondents were interviewed using theme-based questionnaires.

### 3.2. Study Setting

Phase 1 was conducted from May 2021 to July 2022 at all PUBS and PRIVS healthcare facilities that were reporting on SVS and EVDS [14]. Phase 1 included the Pfizer rollout, which began in May 2021, and the J&J rollout, which commenced in June 2021 in KZN. Phase 2 interviews were conducted online via Microsoft Teams from January to March 2024.

### 3.3. Sampling Strategy

*Phase 1:* A total population sampling strategy was employed, including all facilities and districts identified in the dataset, thereby eliminating the need for sample selection. Representativeness across urban and rural settings was inherently achieved, as all 11 districts (A, B, C, D, E, F, G, H, I, J, K), encompassing both urban and rural areas, were included.

*Phase 2:* Vaccination managers who were coordinating C19V supply management were selected from the PUBS and PRIVS. Twelve and eight interview invitations were sent to PUBS and PRIVS vaccination managers, respectively. One vaccination manager from each PUBS district, the PUBS Provincial Pharmaceutical Services, and each PRIVS organisation was invited to participate. Only managers who provided consent by completing the consent form were included; those who did not consent were excluded. Participants were anonymized and interviews were confidential. Interviews were comprehensive, covering aspects of the vaccine supply chain, including planning and coordination, legislative requirements, training, human resources, wastage minimisation, cold chain management, adverse events, transportation, rural area challenges, successes, weaknesses, barriers, risks, risk mitigation, and public–private partnerships. For the purposes of this paper, only stock management and monitoring were considered.

### 3.4. Data Collection

*Phase 1:* Issued doses of Pfizer and J&J were obtained from the SVS web portal, while administered doses were retrieved from the EVDS [14]. Data were aggregated monthly and consolidated in an Excel spreadsheet for each facility, district, and KZN Province, detailing the administered and issued doses of Pfizer and J&J COVID-19 vaccines. Pfizer data were collected from 1 May 2021 to 31 July 2022 (15 months), and J&J data were collected from 1 June 2021 to 31 July 2022 (14 months).

*Phase 2:* Vaccination managers received the interview questions upon providing consent. Interviews were conducted, recorded, and transcribed. A semi-structured interview guide ensured consistency, and clarifications and real-time member validation enhanced accuracy. All participants were fully informed about the anonymity of interviews and the reasons for the research, and how their data would be used. Due to variations in the COVID-19 vaccine supply chain experience between urban, rural districts, and public and private sectors, thematic saturation was not used to determine sample adequacy. The aim was to capture a breadth and diversity of perspectives across different contexts.

### 3.5. Data Analysis and Validation

*Phase 1:* Vaccine usage rates (VURs) [1] were calculated at the KZN provincial level by adding the total number of administered and issued doses for Pfizer and J&J across all districts, then dividing the number of administered doses by the number of issued doses and expressing the value as a percentage. Analyses were conducted collectively for KZN and separately for grouped PUBS and PRIVS healthcare facilities. District-level VURs were similarly calculated for all facilities within each district. VURs exceeding 100% indicated that administered doses exceeded the issued doses, likely due to the non-reporting of issued doses. Since this data was only reviewed approximately two years after the programme, missing data could not be accessed as the programme had already ended, and the SVS COVID-19 application was no longer functional. However, during Phase 1, SVS and EVDS were programmatic data systems that were reliant on facility-level reporting. Therefore, data validation was performed at the facility, district, and provincial levels.

*Phase 2:* Several strategies ensured the validity of qualitative data. Credibility was enhanced as respondents confirmed emerging interpretations during interviews. Dependability was supported through an audit trail maintained in NVivo and Excel. Transferability was promoted by systematically comparing themes between respondents from the public and private sectors.

### 3.6. Statistical Analysis

*Phase 1: Quantitative Study:* Phase 1 data represented vaccine usage rates rather than raw counts. Rates were calculated relative to total administered doses within PUBS and PRIVS, allowing direct comparison across the two groups. Before performing the independent samples t-tests, the underlying distribution of the usage rates was examined using the Kolmogorov–Smirnov test and the Shapiro–Wilk test, alongside the visual inspection of Q–Q plots, which indicated that the data were sufficiently close to normal to justify the use of parametric methods. Furthermore, a test of a hypothesis about the mean was conducted, and according to the central limit theorem, the mean was also normally distributed.

*Phase 2: Qualitative Study:* An inductive thematic content analysis approach was applied. This process involved systematically identifying, analysing, and reporting patterns within the interview transcripts without imposing pre-existing coding frames or theoretical perspectives. The analysis began with open coding, where meaningful segments of text were highlighted and assigned descriptive codes. These codes were then reviewed, compared, and grouped into broader categories to capture underlying ideas. Through an iterative process, categories were refined and consolidated into themes that reflected recurring and non-recurring patterns. NVivo software Version 15 facilitated transcript management, coding, and organisation, but the identification and interpretation remained researcher-driven. Themes were compared across PUBSR and PRIVSR to ensure consistency and diversity, catalogued in Excel, and aggregated as proportions of total PUBSR and PRIVSR.

### 3.7. Ethical Approval

The study received ethics approval from the University of KwaZulu-Natal Ethics Committee (Ref. No.: BREC 4505/2022), the KwaZulu-Natal Department of Health (Ref. No.: KZ_202208_035), and the private sector healthcare authorities.

## 4. Results

### 4.1. Quantitative Analysis

#### Provincial VUR Analysis: Pfizer

KZN Province issued and administered a total of 4,763,232 and 3,897,573 Pfizer doses in 11 PUBS and 6 PRIVS districts, respectively, recording a total provincial VUR of 81.8%. Pfizer PUBS vaccinations were noted in a maximum of 511 vaccination facilities, with only 193 (39%) facilities reporting VUR. Meanwhile, Pfizer PRIVS stated a maximum of 40 facilities, of which only 29 (72.5%) recorded VUR.

Pfizer PUBS and PRIVS issued 87.7% and 12.3% of the doses from pharmacy/bulk storage (Table 1), while administering 84.4% and 15.6% of the doses, respectively. A further analysis of the total provincial Pfizer VUR showed that Pfizer PUBS and PRIVS recorded a VUR of 78.7% (*n* = 73–193) and 104,4% (*n* = 1–29), respectively. Pfizer PUBS VUR was mostly calculated in clinics (*n* = 47–116), followed by hospitals (*n* = 54–62). PRIVS Pfizer VUR was primarily calculated at retail pharmacies (*n* = 8–23), medical aids (*n* = 1–2), and private hospitals (*n* = 1–4).

**Figure 1:**
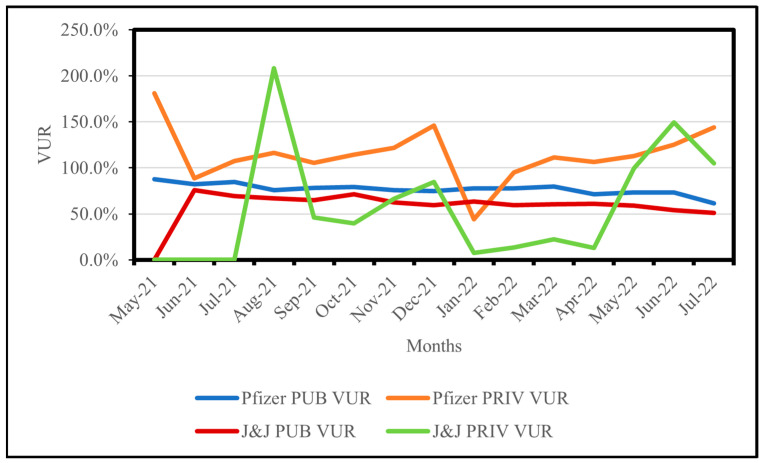
Monthly vaccine usage rates (VURs) for Pfizer and Janssen (J&J) in public (PUBS) and private (PRIVS) sectors, KwaZulu-Natal, South Africa, May 2021–July 2022. Note: VUR > 100% indicates administered doses exceeded issued doses due to under-reporting.

Table 1 and Figure 1 demonstrate that in July 2021, Pfizer PUBS documented the maximum number of issued and administered doses, accompanied by a VUR of 84.6% (*n* = 82). However, the maximum VUR of 87.8% (*n* = 73) was recorded in May 2021. A decrease in Pfizer PUBS VUR occurred from March 2022 onwards, culminating in the lowest recorded VUR of 61.6% (*n* = 168) in July 2022. The maximum number of VUR reporting facilities was observed in May and June 2022 (*n* = 193), yielding VURs of 73.3% and 73.4%, respectively.

Pfizer PRIVS VUR exceeded 100% for 13 (87%) months of study period and fluctuated considerably. As shown in Table 1 and Figure 1, Pfizer PRIVS recorded the highest VUR of 181.2% (*n* = 1) in May 2021. By August 2021, the highest number of PRIVS doses issued and administered was recorded, with a VUR of 116.5% (*n* = 24). December 2021 recorded a VUR of 146.1% (*n* = 25), which decreased to 44% (*n* = 25) in January 2022.

### 4.2. District VUR Analysis: Pfizer

**Figure 2:**
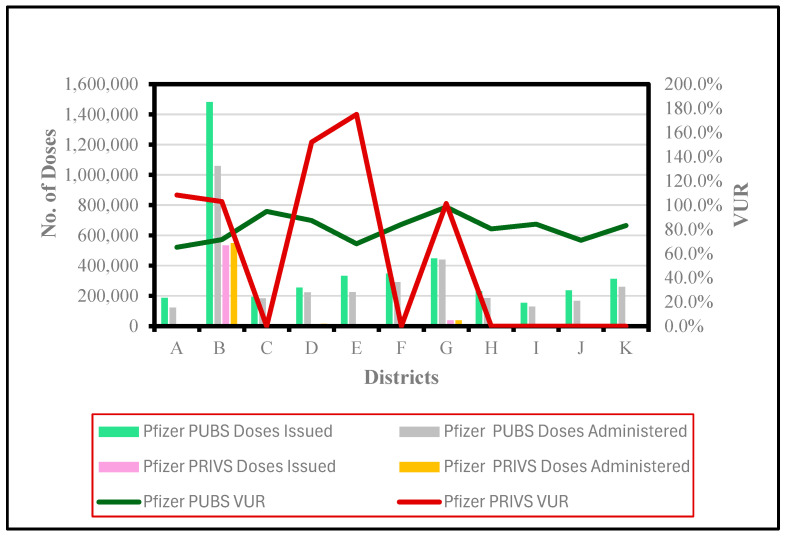
Issued and administered Pfizer vaccine doses and corresponding vaccine usage rates (VUR) in public (PUBS) and private (PRIVS) sectors across KwaZulu-Natal districts, May 2021–July 2022. Note: VUR > 100% indicates doses administered exceeded doses issued due to under-reporting.

Pfizer VUR was calculated in 100% of (*n* = 11) PUBS districts for the study period (Figure 2). Figure 2 shows that Pfizer PUBS District B recorded a VUR of 71.4% (*n* = 21–23), with the maximum number of doses issued and administered. Conversely, Pfizer PUBS District G exhibited the highest VUR of 98.4% (*n* = 7–9). Pfizer PUBS District K reported the highest number of VUR facilities (*n* = 33–62) with a VUR rate of 83.1%.

Only six districts (A, B, D, E, F, G) provided Pfizer vaccinations in PRIVS, while VUR was only calculated (A, B, D, E, G) for five districts. Figure 2 indicates that PRIVS District B (*n* = 12–23) recorded a VUR of 103%, with the highest number of issued and administered doses. However, the highest VUR of 175% was observed in District E (*n* = 1), with minimal doses issued and administered. 

Statistical significance (*p* < 0.05) was noted between Pfizer PUBS and PRIVS VUR for 66.7% (10 months, from July to November 2021, February to April 2022, and June to July 2022) of SP.

### 4.3. Provincial VUR Analysis: J&J

KwaZulu-Natal (KZN) Province issued and administered a total of 2,078,598 and 1,314,954 J&J doses in 11 and 6 districts in PUBS and PRIVS, respectively, yielding a total provincial VUR of 63.3%. Although a maximum of 603 PUBS and 43 PRIVS facilities administered J&J, only 184 (30.5%) PUBS and 16 (37.2%) PRIVS facilities reported VUR. Table 1 shows that PUBS predominated in J&J vaccinations, issuing 94% and administering 96% of the doses. A deeper analysis of provincial VUR revealed that J&J PUBS and PRIVS recorded VURs of 64.7% (*n* = 49–184) and 40.2% (*n* = 4–16), respectively. J&J PUBS VUR was predominantly calculated in clinics (*n* = 48–107), followed by hospitals (*n* = 36–62). J&J PRIVS VUR was primarily calculated at retail pharmacies (*n* = 4–12), medical aids, and private hospitals (*n* = 1–2).

Table 1 and Figure 1 show that in August 2021, J&J PUBS recorded the highest number of issued and administered doses, with a VUR of 66.7% (*n* = 81). However, the maximum PUBS VUR of 75.9% (*n* = 49) was noted in June 2021, before declining to a minimum of 50.8% (*n* = 151) by July 2022 (Table 1 and Figure 1).

For PRIVs, Table 1 and Figure 1 illustrate that in August 2021, J&J PRIVS (*n* = 5) documented the highest VUR of 208%, while in November 2021, a VUR of 66.2% (*n* = 16) was recorded with the maximum number of administered doses. January 2022 documented the maximum number of issued doses with a VUR of 7.4% (*n* = 14).

### 4.4. District VUR Analysis: J&J

**Figure 3:**
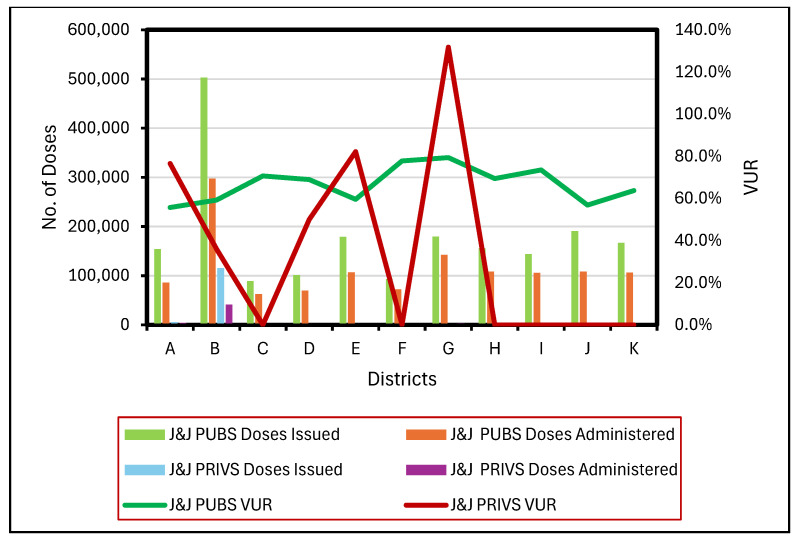
Issued and administered Janssen (J&J) vaccine doses and corresponding vaccine usage rates (VUR) in public (PUBS) and private (PRIVS) sectors across KwaZulu-Natal districts, May 2021–July 2022. Note: VUR > 100% indicates administered doses exceeded issued doses due to under-reporting.

J&J PUBS VUR were calculated in all 11 districts (100% SP). PUBS District B (*n* = 11–24) recorded the highest number of issued and administered doses yet reported a VUR of 59.2% (Figure 3). Conversely, District G (*n* = 5–9) exhibited the highest VUR of 79.4%. District K (*n* = 6–54), with the greatest number of facilities, recorded a VUR of 63.7%.

J&J PRIVS vaccinations were conducted in six districts. However, J&J PRIVS VUR was calculated for five districts (A, B, D, E, and G). District B (*n* = 2–13) reported the maximum number of issued and administered doses but also recorded the lowest overall VUR 36%. In contrast, District G (*n* = 1) reported the highest VUR of 131.8%. 

Statistical analysis revealed a significant difference (*p* < 0.05) between J&J PUBS and PRIVS VUR for 21% of the study period (three months: August 2021, June 2022, and July 2022).

### 4.5. Qualitative Analysis:

#### 4.5.1. Stock Monitoring

Twelve PUBSR (100%) and seven PRIVSR (87.5%) were interviewed between January and March 2024.

Electronic stock management systems (100%, *n* = 12 PUBSR; 100%, *n* = 7 PRIVSR), manual stock management systems (67%, *n* = 8 PUBSR; 43%, *n* = 3 PRIVSR), and stock management staff (83%, *n* = 10 PUBSR; 57%, *n* = 4 PRIVSR) were examined. Stock monitoring was facilitated through SVS reporting (100%, *n* = 12 PUBSR; 100%, *n* = 7 PRIVSR), web monitoring (17%, *n* = 2 PUBSR), coordinated reporting systems (67%, *n* = 8 PUBSR; 100%, *n* = 7 PRIVSR), and reporting staff (25%, *n* = 3 PUBSR; 14%, *n* = 1 PRIVSR).

Other reported practices included the use of manual templates for SVS and EVDS input (25%, *n* = 3 PUBSR; 29%, *n* = 2 PRIVSR), centralised SVS capturing (14%, *n* = 1 PRIVSR), SVS desktop connectivity challenges and use of mobile devices (8%, *n* = 1 PUBSR), and stock transfer reporting challenges were recorded (8%, *n* = 1 PUBSR; 29%, *n* = 2 PRIVSR).

Daily (100%, *n* = 12 PUBSR; 100%, *n* = 7 PRIVSR) and hourly C19V reconciliations (29%, *n* = 2 PRIVSR) were noted, as well as the use of reconciliation indicators (42%, *n* = 5 PUBSR; 57%, *n* = 4 PRIVSR), reconciliation challenges (25%, *n* = 3 PUBSR; 29%, *n* = 2 PRIVSR), and non-non-integration of SVS and EVDS (14%, *n* = 1 PRIVSR) were cited.

#### 4.5.2. Monitoring and Evaluation

Monitoring and evaluation (M&E) began at the onset of C19V programme (100%, *n* = 12 PUBSR; 43%, *n* = 3 PRIVSR) and was ongoing (100%, *n* = 12 PUBSR; 57%, *n* = 4 PRIVSR). Daily (42%, *n* = 5 PUBSR; 43%, *n* = 3 PRIVSR) meetings occurred with data review and analysis (58%, *n* = 7 PUBSR; 43%, *n* = 3 PRIVSR).

Programmatic indicators included clients vaccinated (100%, *n* = 12 PUBSR; 71%, *n* = 5 PRIVSR), home-based care clients (14%, *n* = 1 PRIVSR), and adverse events (42%, *n* = 5 PUBSR; 14%, *n* = 1 PRIVSR). Operational indicators included EVDS, SVS reporting (43%, *n* = 3 PRIVSR), human resources management, financial/billing, and queue management 43%, *n* = 3 PRIVSR). C19V stock indicators included stock on hand (58%, *n* = 7 PUBSR; 71%, *n* = 5 PRIVSR), wastages and expiry dates (58%, *n* = 7 PUBSR), short-dated stock (42%, *n* = 5 PUBSR), and overall vaccine supply management (100%, *n* = 12 PUBSR; 100%, *n* = 7 PRIVSR).

## 5. Discussion

Our analysis revealed that the total provincial VUR of 81.8% for Pfizer and 63.3% for J&J, provided a broad view of C19V supply management in KZN. However, a PUBS and PRIVS analysis was further required to understand the insights into the distribution, administration, and reporting aspects of the C19VSC and C19V uptake between both these sectors. Pfizer PUBS and PRIVS recorded a VUR of 78.7% and 104.4%, respectively. However, J&J PUBS and PRIVS recorded lower VURs of 64.7% and 40.2%, respectively. Although PUBS administered the majority of Pfizer (84.4%) and J&J (96.2%) C19V doses, PRIVS participation improved overall C19V coverage and contributed to advancing public health and health equity goals [15]. KZN issued nearly double the number of Pfizer C19V doses and administered three times more Pfizer C19V doses relative to J&J. However, only six districts administered Pfizer and J&J in PRIVS, as smaller facilities in the remaining five districts could not afford the high cost of full vaccine trays [16]. PUBS and PRIVS facilities were predominantly clinics and retail pharmacies, with only one private doctor participating in PRIVS.

Pfizer, with a 95% efficacy and strong safety profile [17], was the preferred vaccine in KZN, recording PUBS and PRIVS VURs of 78.7% and 104.4%, respectively. Due to its specialised storage requirements [18], the Pfizer vaccine was administered at fewer facilities (511 PUBS, 40 PRIVS) compared to J&J. NDOH’s minimum standards mandated that all C19V storing facilities must possess Section 22A (15) permits and report stock daily via SVS [14]. Section 22A (15) permits were issued to facilities that fulfilled the long term storage criteria and were performing vaccinations. However, PUBS mobile and fixed outreach sites did not fulfil the long term storage criteria and were not issued Section 22A (15) permits. Therefore, these PUBS mobile and fixed outreach sites lacked Section 22A(15) permits did not report stock issue data directly on SVS, but only recorded C19V administered on EVDS. As a result, VURs could not be calculated for outreach facilities due to missing stock issue data, which in turn contributed to the reduced number of Pfizer and J&J PUBS facilities reporting VUR.

However, doses issued to outreach facilities were reported on SVS by the distribution facilities and were included in each district’s total stock issued. District-level VUR for Pfizer PUBS ranged from 71 to 88% across 93% of SP, indicating that doses administered were consistently lower than the doses issued. KZN includes many rural districts [19], with distant outreach facilities; therefore, distribution sites routinely provided additional doses for community clients not registered on the EVDS to maximise coverage. This practice, however, increased the risk of C19V wastage and unreported losses [20]. From May (VUR 87.8%) to June 2021 (VUR 82.1%), KZN emerged as the leading SA Province in administering Pfizer vaccinations [21].

In July 2021, Pfizer PUBS recorded the maximum number of doses issued and administered, despite political unrest, riots, and the closure and looting of vaccination sites [22]. Pfizer PUBS maintained VUR values of 70–79%, even as the number of reporting facilities increased (*n* = 73–193), suggesting more consistent and skilled SVS reporting compared with PRIVS, as PUBS adopted SVS since 2016 [23].

Pfizer PRIVS facilities reporting VUR accounted for 72.5%, nearly double that of J&J. For 80% of SP, Pfizer PRIVS reported VUR > 100% (*n* = 1–29), indicating that doses administered exceeded issued doses, largely due to non-reporting of issued doses on SVS and suboptimal monitoring of reporting. During January 2022 (*n* = 25), Pfizer PRIVS VUR decreased substantially to 44% (*n* = 25), as issued doses were double the administered doses. This reflected a lack of forecasting and highlighted the risk of wastage, particularly given Pfizer’s limited shelf-life after changes to storage conditions [18].

During the August 2021 J&J rollout to educators [24], PUBS reported the maximum number of issued and administered doses, with a VUR of 66.7%, while PRIVS recorded a maximum VUR of 208%. From October 2021 onwards, the number of PUBS clinics increased following the acquisition of Section 22A (15) permits [14], leading to fluctuations of 50–60% in PUBS VUR. For six months of SP, J&J PRIVS recorded a VUR < 50%, highlighting that Pfizer (Table 1) was preferred in PRIVS and that vaccine hesitancy contributed to reduced uptake in KZN [25]. In January 2022, J&J PRIVS (similar to Pfizer PRIVS) recorded its lowest VUR of 7.4%, highlighting that the issued stock greatly exceeded administered stock and a lack of forecasting-based issuing, which may have been perpetuated by untrained locum staff.

In District B, a densely populated urban area, neither Pfizer nor J&J in PUBS or PRIVS achieved the highest VUR, despite administering and issuing the greatest number of doses due to reporting challenges. PUBS District K, with the highest number of facilities, reported VURs of 83.1% (Pfizer) and 63.7% (J&J), respectively. This can be attributed to consistent SVS reporting and enhanced vaccination uptake strategies [26]. PRIVS facilities in districts F and G did not record VURS due to non-reporting of issued doses on SVS. This gap, unaddressed by PRIVS managers, posed a risk to future reimbursement processes. These findings align with Mbonane et al., who reported that continuous training of healthcare workers can improve SVS reporting [23]. Our study similarly recommended that PRIVS managers monitor stock reporting on the recommended NDOH systems to reduce reconciliation challenges during reimbursement.

The use of three separate systems—electronic stock management systems, stock cards, and SVS—introduced risks of non-reporting due to duplication of data capture. Although PRIVSR reported a higher use of coordinated SVS reporting systems, PRIVS overall showed inconsistent SVS reporting for Pfizer and J&J, as evidenced by frequent instances of administered doses exceeding issued doses. During network disruptions, PUBSR and PRIVSR used manual data-capture forms to mitigate data loss. One PRIVSR noted that the head office conducted SVS capturing for all sites, which ensured reporting but limited opportunities for site-level staff to gain experience in NDOH medication surveillance systems.

Both PUBSR and PRIVSR undertook reconciliations, but methods varied between these sectors and were complicated by stock transfers. This underscores the need for electronic reconciliation systems. SA’s connectivity challenges [26] likely exacerbated SVS reporting delays. Expansion of cell tower infrastructure in rural areas, as recommended by Jiluwane [27], is expected to improve connectivity but requires prioritisation and coordination by the government and operators.

PUBSR stressed that failure was not an option during the C19V programme, leading to structured, continuous monitoring through daily meetings focused on progress, challenges, and solutions within the C19V programme. While vaccinated clients and C19V stock were common PUBS and PRIVS indicators for forecasting, vaccine hesitancy [25] resulted in an unpredictable demand and C19V stockpiling. PRIVSR focused on financial feasibility, staff performance, and efficient queue management to reduce waiting times. PUBS demonstrated greater vigilance than PRIVS in monitoring C19V wastage and redistributing short-dated stock to minimise stock loss.

## 6. Conclusions

This study recommends that PUBS and PRIVS collaboratively and proactively finalise comprehensive service-level agreements and reimbursement policies in preparation for future pandemics/immunisation programmes. Given SAs high burden of HIV and TB [28], it is essential to identify strategies and interventions that strengthen PUBS–PRIVS partnerships for sustainable collaboration. Such partnerships can bring together complementary resources and expertise to support both communicable and non-communicable disease programmes, thereby synergistically advancing public health and equity [14], as demonstrated during the C19V programme.

While SVS and EVDS online training introduced both new successes and challenges, our findings support the need for enhanced operational training of staff, including virtual workshops, phone-based consultations, implementation-focused video calls, and structured training evaluation, as recommended by Worley et al. [29]. Furthermore, PUBS and PRIVS managers should adopt robust monitoring and evaluation processes, implement standardised data review systems, and leverage artificial intelligence-based methodologies to improve data quality and monitor usage and wastage of vaccines [30].

Our study retrospectively calculated VUR by manually merging EVDS and SVS datasets. Notably, the highest VURs were not always associated with the highest numbers of issued or administered doses, nor with the number of facilities reporting. A critical barrier identified by this study was the lack of integration between EVDS and SVS, which prevented real-time automated VUR calculation. Integration of these systems could involve developing interoperable platforms for automatic data sharing, standardising reporting protocols, conducting regular reconciliation audits, training facility staff on integrated data entry, and establishing real-time monitoring dashboards.

Vaccine procurement costs comprise a significant share of immunisation programme costs in low- and middle-income countries, yet not all procured vaccines are administered [31]. Additionally, lower vaccination uptake increases morbidity and mortality, adding to healthcare costs. System integration could therefore serve as a long-term cost-saving measure, supported through a combination of national immunisation budgets, donor funding, and cost-sharing with private technology providers.

This study also recommends that all vaccination sites capture both stock issued and stock administered for real-time calculation of VURs for prompt intervention. Real-time VUR could signal reporting gaps, identify supply chain inefficiencies, highlight district-level vaccine uptake and losses, and inform PRIVS reimbursements. Best practices from districts with consistently high VUR should be shared across health districts. As Lazarus et al. noted, accurate national reporting of vaccine wastage rates remains limited [20]. Our study proposes extrapolating vaccine wastage rates from VUR as a proactive strategy to inform vaccine waste reduction initiatives [1].

This study recommends the development of a single, national, integrated vaccination record system that combines client-level data, vaccine stock information, and adverse event reporting [32]. Such a system would provide daily automated VUR calculations, support evidence-based decision-making, and proactively address stock management challenges. It would also eliminate the need for paper-based records, particularly in the context of travel, childhood vaccinations, and pandemic vaccinations. Furthermore, this system could also be used to access details (batch numbers and expiry dates) of vaccines when adverse events are reported, treated, and causality analysis is undertaken.

This study aligns with the findings of Gengiah et al. [25], which suggests that health authorities should prioritise specific vaccine-hesitant groups using targeted information campaigns, social media, and digital channels to enhance vaccine uptake [24]. Empowering traditional, religious, and political leaders to promote vaccine acceptance may reduce hesitancy, particularly in rural KZN communities [24].

Finally, this study, informed by WHO’s [1] and South Africa’s monitoring systems [14], extends beyond descriptive reporting to understanding successes, challenges, and opportunities for improvement. This research enhances theoretical understanding by demonstrating the impact of supply chain integration, monitoring, and workforce practices on vaccine availability and acceptance. From a policy perspective, the study provides evidence to inform interventions such as integrating stock management and patient administration/dispensing systems, optimising training, and improving monitoring systems, which may enhance the efficiency and effectiveness of current and future vaccination programmes both nationally and in similar low- and middle-income contexts.

## 7. Limitations

This study has several limitations. First, non-reporting on SVS and EVDS resulted in missing stock and vaccination data. While manual templates were used as backups, they were used particularly in PUBS. Second, although the qualitative sample was small, it provided rich, in-depth, hands-on perspectives from vaccination managers from rural and urban districts of PUBS and PRIVS in resource-constrained KZN. These lessons remain valuable for informing future vaccination programmes. Finally, outreach sites that lacked Section 22A (15) permits did not store vaccines on-site and therefore did not report issued stock directly into the SVS. Instead, distributing facilities captured stock data on their behalf. Our study is recommending that all vaccination sites record stock issued and administered; thereby enabling the real-time electronic calculation of VURS. These perspectives are crucial for shaping future vaccination strategies.

## Figures and Tables

**Table 1:** Monthly Pfizer and Janssen (J&J) COVID-19 vaccine doses issued, doses administered, vaccine usage rates (VURs), and number of reporting facilities in public (PUBS) and private (PRIVS) sectors, KwaZulu-Natal, South Africa, May 2021–July 2022.

Month	Pfizer PUBS: Doses Issued	Pfizer PUBS Doses Administered	Pfizer: PUBS VUR	Pfizer PUBS: VUR Reporting Facilities (*n*%) *	Pfizer PRIVS: Doses Issued	Pfizer PRIVS: Doses Administered	Pfizer PRIVS: VUR	Pfizer PRIVS: VUR Reporting Facilities (*n*%) *	J&J PUBS: Doses Issued	J&J PUBS: Doses Administered	J&J PUBS: VUR	J&J PUBS: VUR Reporting Facilities (*n*%) *	J&J PRIVS: Doses Issued	J&J PRIVS: Doses Administered	J&J PRIVS: VUR	J&J PRIVS: VUR Reporting Facilities (*n*%) *
May-21	239,208	209,953	87.8%	73 (42.9%)	1476	2675	181.2%	1 (5.6%)	0	0	0	0	0	0	0 ^1^	0%
Jun-21	333,534	273,900	82.1%	80 (25.2%)	21,006	18,633	88.7%	12 (57.1%)	103,872	78,862	75.9%	49 (36.3%)	0	6	0 ^1^	0%
Jul-21	631,944	534,937	84.6%	82 (25.9%)	53,682	57,723	107.5%	19 (55.9%)	215,286	149,240	69.3%	79 (35.6%)	0	6668	0.0%	0%
Aug-21	402,984	304,513	75.6%	82 (27.6%)	117,606	136,984	116.5%	24 (64.9%)	334,770	223,305	66.7%	81 (27.6%)	3312	6890	208.0%	5 (38.5%)
Sept-21	451,650	353,092	78.2%	84 (25.1%)	110,082	115,870	105.3%	27 (73%)	183,084	118,701	64.8%	77 (26.4%)	12,522	5758	46.0%	6 (60%)
Oct-21	397,476	315,006	79.3%	84 (21.9%)	93,336	106,718	114,3%	29 (72.5%)	214,716	152,639	71.1%	80 (24.2%)	10,320	4092	39.7%	5 (31,3%)
Nov-21	296,760	224,469	75.6%	130 (30.9%)	35,970	43,842	121.9%	26 (65%)	205,986	128,623	62.4%	131 (33.8%)	17,790	11,770	66.2%	16 (61.5%)
Dec-21	194,946	146,076	74.9%	152 (36.1%)	18,546	27,096	146.1%	20 (51.3%)	115,902	68,634	59.2%	150 (39%)	5214	4426	84.9%	10 (38.5%)
Jan-22	207,918	162,031	77.9%	157 (37.1%)	67,062	29,486	44.0%	25 (67.6%)	111,966	70,954	63.4%	146 (33.8%)	34,440	2561	7.4%	14 (60.9%)
Feb-22	205,836	159,588	77.5%	182 (39.1%)	18,438	17,490	94.9%	24 (77.4%)	114,456	67,996	59.4%	178 (29.5%)	19,578	2664	13.6%	12 (66.7%)
Mar-22	252,036	201,198	79.8%	185 (37.4%)	21,372	23,817	111.4%	20 (64.5%)	112,836	68,228	60.5%	177 (37.4%)	12,342	2776	22.5%	11 (61.1%)
Apr-22	146,796	104,848	71.4%	172 (62.5%)	10,032	10,667	106.3%	18 (72%)	62,148	37,868	60.9%	152 (34.4%)	7644	1008	13.2%	8 (53.3%)
May-22	220,788	161,804	73.3%	193 (37.9%)	6774	7656	113.0%	17 (63%)	89,610	52,669	58.8%	184 (38.5%)	762	758	99.5%	6 (54.5%)
Jun-22	125,994	92,446	73.4%	193 (39.1%)	5430	6808	125.4%	14 (56%)	56,130	30,231	53.9%	177 (39%)	270	403	149.3%	4 (36.4%)
Jul-22	71,736	44,195	61.6%	168 (37.8%)	2814	4052	144.0%	12 (57.1%)	33,420	16,991	50.8%	151 (38%)	222	233	105.0%	4 (36.4%)
Total	4,179,606	3,288,056	78.7%		583,626	609,517	104.4%		1,954,182	1,264,941	64.7%		124,416	50,013	40.2%	

Note: (*n*%) refers to the number and percentage of facilities that reported VUR within each month; 0 = no stock was issued or administered; values exceeding 100% indicate administered doses greater than issued dose; due to non-reporting of issued doses. 0—indicates that no stock was issued or administered. Private Sector: PRIVS.; Public Sector: PUBS. Vaccine Usage Rate: VUR. District VUR Analysis: Pfizer. * (*n*%) refers to the number and percentage of facilities that reported VURs for each month. Percentage was calculated by the number of VUR reporting facilities divided by the sum of VUR reporting and VUR non-reporting facilities. ^1^ refers to J&J doses administered.

## Data Availability

The data supporting the findings of this study are available from the corresponding author upon reasonable request.

## Acronyms

COVID-19	C19
COVID-19 Vaccine	C19V
Private Sector	PRIVS
Private Sector Respondents	PRIVSR
Public Sector	PUBS
Public Sector Respondents	PUBSR
Study Period	SP
Vaccine Usage Rate	VUR
National Department of Health	NDOH
Stock Visibility System	SVS
Electronic Vaccination Data System	EVDS
